# 4D tissue phase mapping: clinically viable acquisition protocol and new method of visualisation

**DOI:** 10.1186/1532-429X-17-S1-O46

**Published:** 2015-02-03

**Authors:** Robin Simpson, Marius Menza, Juergen Hennig

**Affiliations:** 1 Medical Physics, University Medical Centre, Freiburg, Germany

## Background

Tissue phase mapping (TPM) has been shown to be capable of providing insights into healthy and diseased motion [1]. Typically 3 short-axis slices are acquired, providing full 3-directional velocity vectors but only in 2D slices, preventing the calculation of potentially important parameters such as through-plane strain rate. More recently, several studies have presented 4DTPM [2,3], however acquisition times can be unfeasibly long, image quality, temporal and spatial resolution is generally poor, and visualising the data is a challenge. This abstract presents initial application of EnSight (CEI, USA) visualisation to 4DTPM data, acquired with a duration, quality and resolution which do not prevent clinical translation.

## Methods

A navigator-gated black-blood TPM sequence [4] was adapted to allow 3D acquisitions. A 3D slab with matrix size 120x160x8 (75% slice oversampling) was acquired with spatial resolution of 2x2x4mm and temporal resolution of 24.4ms (40 phases) in a healthy volunteer. PEAK-GRAPPA [5] factor 5 led to acquisition time of 16m45s. Images were manually segmented using MATLAB (The Mathworks, MA) before importing into EnSight. Vector arrows were used at each pixel to represent speed at each position.

## Results

**Figure 1:**
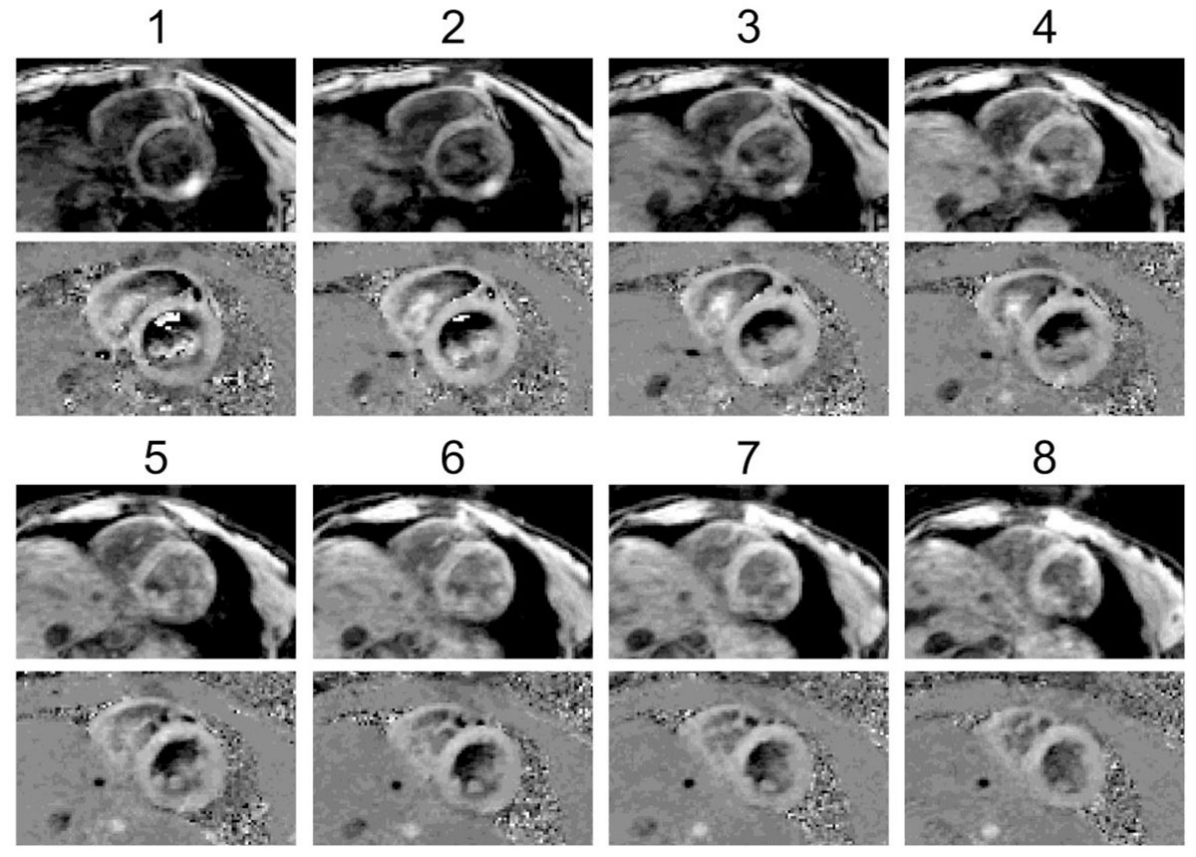
Magnitude (top) and through-plane velocity images (bottom) in all slices during diastole.

**Figure 2:**
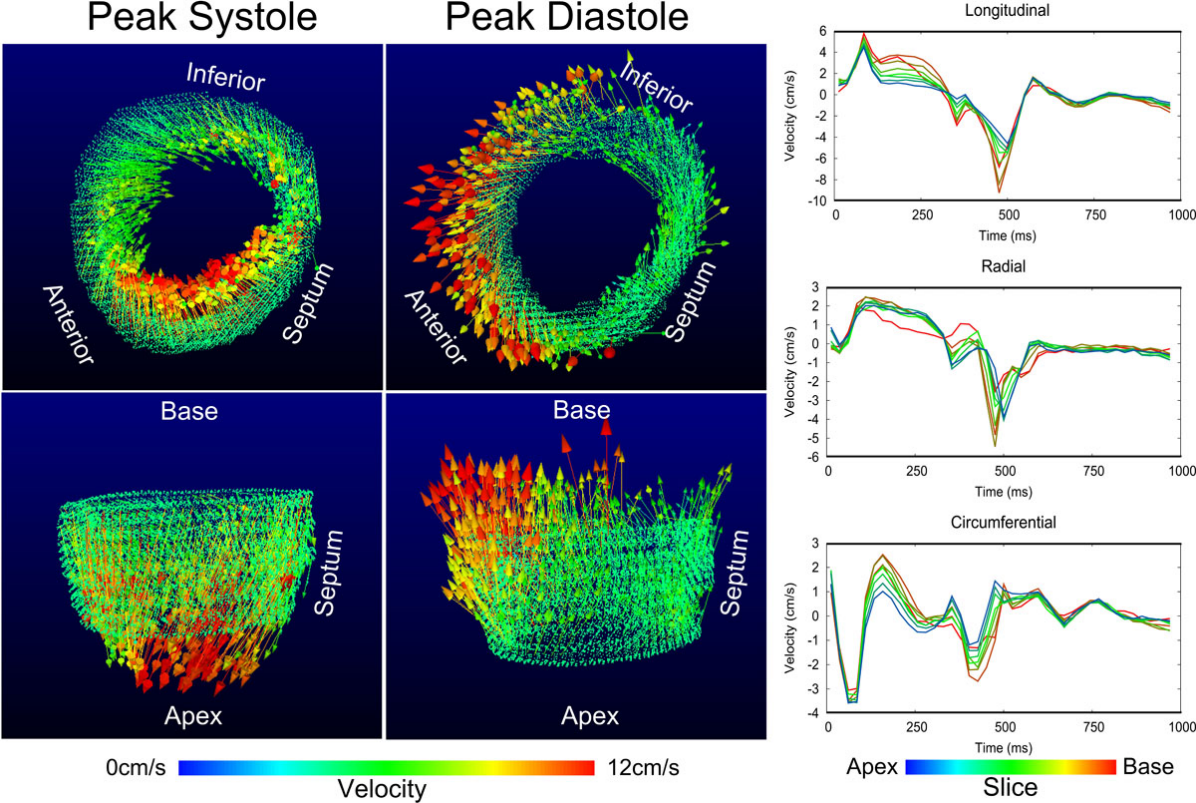
Left: 3D visualisations of velocities shown at two points in the cardiac cycle. Top images show a short-axis view from a basal position, bottom images show the ventricle viewed from the antero-septum. Arrows show the velocity at each position colour-coded according to speed. Regional variation in velocities can be seen, as can the reversal of velocity directions between peak systole and peak diastole (for example clockwise motion at peak systole and anti-clockwise motion at peak diastole). Right: Velocity-time curves for all slices. Velocities are decomposed into longitudinal, radial and circumferential directions according to a co-ordinate system based on the left ventricle. The line colour corresponds to slice position as shown by the colour bar beneath. Velocities from the most basal slice appear to be affected by image artefacts, possibly due to saturation effects.

Image quality in all slices can be seen in Figure 1 where magnitude and through-plane phase images during diastole are shown. Figure 2 shows EnSight visualisations of the 3D volume from two views at peak systole and peak diastole, along with velocity-time curves for all slices. EnSight visualisations clearly show regional variation of velocities, for example lowered septal peak diastolic velocities. The graphs show expected motion features, including the notch in radial and circumferential velocities due to isovolumic relaxation. A clear decrease in peak velocities (eg peak diastolic longitudinal velocity) from base to apex can also be seen.

## Conclusions

By adapting a 2DTPM sequence, high quality 4DTPM data can be acquired in a scan time that does not prevent clinical use. In-plane and temporal resolution are high, while through plane resolution was sacrificed in favour of acquisition time (it is not expected that this will greatly affect derived parameters). A limitation of the acquisition is that the entire ventricle is not covered. The protocol must therefore be adapted to acquire the pertinent data in future studies. EnSight allows visualisation of 4D data which can provide intuitive understanding of the motion occurring throughout the cardiac cycle. While only two time frames and two views are shown here, in reality the motion can be viewed as a 3D cine which can be interactively rotated in all dimensions. Future acquisitions in patients will highlight the use of such visualisation and may provide a better understanding of the complex cardiac motion.

## Funding

N/A.
